# Molecular Portrait of Oral Tongue Squamous Cell Carcinoma Shown by Integrative Meta-Analysis of Expression Profiles with Validations

**DOI:** 10.1371/journal.pone.0156582

**Published:** 2016-06-09

**Authors:** Soundara Viveka Thangaraj, Vidyarani Shyamsundar, Arvind Krishnamurthy, Pratibha Ramani, Kumaresan Ganesan, Muthulakshmi Muthuswami, Vijayalakshmi Ramshankar

**Affiliations:** 1 Department of Preventive Oncology (Research), Cancer Institute (W.I.A.), Chennai, India; 2 Centre for Oral Cancer Prevention Awareness and Research, Sree Balaji Dental College and Hospital, Chennai, India; 3 Department of Surgical Oncology, Cancer Institute (W.I.A.), Chennai, India; 4 Department of Oral and Maxillofacial Pathology, Saveetha Dental College, Saveetha University, Kumanchavadi, Chennai, India; 5 Department of Genetics, School of Biological Sciences, Madurai Kamaraj University, Madurai, India; Queen Mary University of London, UNITED KINGDOM

## Abstract

Oral Tongue Squamous cell carcinoma (OTSCC), the most frequently affected oral cancer sub-site, is associated with a poor therapeutic outcome and survival despite aggressive multi- modality management. Till date, there are no established biomarkers to indicate prognosis and outcome in patients presenting with tongue cancer. There is an urgent need for reliable molecular prognostic factors to enable identification of patients with high risk of recurrence and treatment failure in OTSCC management. In the current study, we present the meta-analysis of OTSCC microarray based gene expression profiles, deriving a comprehensive molecular portrait of tongue cancer biology, showing the relevant genes and pathways which can be pursued further to derive novel, tailored therapeutics as well as for prognostication. We have studied 5 gene expression profiling data sets available on exclusively oral tongue subsite comprising of sample size; n = 190, consisting of 111 tumors and 79 normals. The meta- analysis results showed 2405 genes differentially regulated comparing OTSCC tumor and normal. The top up regulated genes were found to be involved in Extracellular matrix degradation (ECM) and Epithelial to mesenchymal transition (EMT) pathways. The top down regulated genes were found to be involved in detoxication pathways. We validated the results in clinical samples (n = 206), comprising of histologically normals (n = 10), prospective (n = 29) and retrospective (n = 167) OTSCC by evaluating MMP9 and E-cadherin gene expression by qPCR and immunohistochemistry. Consistent with meta-analysis results, MMP9 mRNA expression was significantly up regulated in OTSCC primary tumors compared to normals. MMP9 protein over expression was found to be a significant predictor of poor prognosis, disease recurrence and poor Disease Free Survival (DFS) in OTSCC patients. Analysis by univariate and multivariate Cox proportional hazard model showed patients with loss of E-cadherin expression in OTSCC tumors having a poorer DFS (HR = 1.566; P value = 0.045) and poorer Overall Survival (OS) (HR = 1.224; P value = 0.003) respectively. Combined over-expression of MMP9 and loss of E-cadherin membrane positivity in the invasive tumor front (ITF) of OTSCC had a significant association with poorer DFS (Log Rank = 16.040; P value = 0.001). These results suggest that along with known clinical indicators of prognosis like occult node positivity, assessment of MMP9 and E-cadherin expression at ITF can be useful to identify patients at high risk and requiring a more intensive treatment strategy for OTSCC. Meta-analysis study of gene expression profiles indicates that OTSCC is a disease of ECM degradation leading to activated EMT processes implying the aggressive nature of the disease. The triggers for these processes should be studied further. Newer clinical application with agents that can inhibit the mediators of ECM degradation may be a key to achieving clinical control of invasion and metastasis of OTSCC.

## Introduction

Oral Tongue Squamous Cell Carcinoma (OTSCC) is regarded as a biologically unique entity compared to cancers occurring in the other oral sub-sites. The trend in epidemiology of oral cancer in Asia in the past decade (2000–2012) shows OTSCC as the most frequently affected oral sub-site. [1] Earlier studies also report a higher incidence of OTSCC in India compared to other countries. [2, 3, 4, 5] According to population based cancer registry (PBCR), the age adjusted incidence rate (AAR) for OTSCC in Chennai is showing an increasing trend from 3.6 to 5.7 per 100,000 persons above 25 years. Though there are poor prognostic indicators for OTSCC like occult node positivity, tumor depth, lymphovascular invasion and perineural invasion, there is still a need for molecular prognostic biomarkers that are reliable and robust to identify patients who are likely to have an adverse outcome.

Microarrays, a tool for genomic scale profiling of gene expression, is a well known potentially valuable means of understanding the complex interactions and networks in development of several diseases including cancer. [6, 7] These high throughput studies have offered the advantage of understanding the biology of a cancer through an exhaustive analysis. The launching of public microarray data archives like Gene Expression Omnibus and the advent of advanced computational informatics tools have made it possible to compare and converge gene expression studies done independently across different platforms. However, the hallmark of scientific progress is reproducibility of published outcomes which has been difficult in the case of several microarray studies with major sources of discordance because of variation caused by random noise, biological and experimental differences, and differences in technical methods. [8] Most often we have findings that are not reproducible across studies due to data perturbations of individual studies, improper validations, and insufficient control of false positives. Despite these obstacles, several groups have successfully gleaned important insights from the focused comparison of disparate microarray results. [9, 10] Many of the limitations can be mitigated by the use of standard reporting methods, together with careful application of large-scale meta-analysis techniques.

Current study presents meta-analysis of OTSCCs as an exclusive sub-site for the first time as our primary objective. It was attempted to overcome the limitations of the individual expression profiling studies, resolving inconsistencies and reducing the likelihood of random errors, thus laying a foundation for uncovering the molecular aspects of OTSCC. We present the differentially expressed genes (DEG) comparing the OTSCC and normal expression profiles along with the involved signaling pathways. We have validated two biomarkers, MMP9 and E-cadherin found in meta–analysis in prospective and retrospective clinical samples as our second objective.

## Materials and Methods

### Identification of eligible OTSCC gene expression data sets

OTSCC expression profiling studies were identified by searching the PubMed database. The following keywords and their combinations were used: “Oral tongue cancer gene expression microarray”. The Gene Expression Omnibus database (http://www.ncbi.nlm.gov/geo) was also searched for terms “Oral Tongue Cancer”, “Oral Tongue Squamous cell carcinoma”, “mobile tongue cancer”.

#### Inclusion Criteria

Gene expression data sets from exclusively anterior 2/3 (mobile tongue cancer) were taken for the study. The original experimental studies comprising of gene expression values for tongue tumor and normal tissues were taken. The expression data sets obtained from only standard microarray platforms were undertaken for the current study.

#### Exclusion Criteria

Studies on head and neck cancer with a few samples on tongue cancer were excluded. Studies from datasets with base of tongue samples, tongue cancer cell lines, non human tissues were excluded. Studies without inclusion of normal samples were excluded.

### Individual Study Analysis

GEO accession number, sample type, platform, number of cases and controls, references and gene expression data were extracted from each of the selected study. The Entrez ID, gene symbol, and gene description were assigned to each probe in the series matrix expression file based on the corresponding platform file. All the expression values were base 2 log transformed. The dChip software was used to remove duplicates for a given Entrez id, by averaging the expression values from multiple probes corresponding to the same gene. [11, 12] The expression values in each data-set were normalized by global median normalization. Each of the data-sets was first analyzed by BRB ArrayTools version 4.4 (http://linus.nci.nih.gov/BRB-ArrayTolls.html) [13] to identify the DEG among the normal and tongue tumour tissue samples. Study specific, gene specific P values were calculated using two sample random permutation t tests. A t statistic (t) for an individual gene was calculated and compared with 10,000 t statistics generated by randomly assigning the sample labels to the expression values of the gene. A transcript was considered differentially expressed when the difference in the mean expression was > 2 fold with a significance of P <0.05. The common genes across 5 data-sets were derived by combined P-value method. To improve cross-batch comparisons, the individual profiles were independently analyzed before combining them. Class comparison by BRB array tools was used to identify the differentially expressed genes between tumor and normal samples in independent studies. Fisher’s χ2-based method was used to calculate the combined P-value for each gene and the average fold- change of the gene across all the data sets were summarized in S1 Table.

### Meta-analysis of the OTSCC data

GEO data tables were constructed from gene-expression data as mentioned earlier with genes/probes in the rows and samples in the columns and uploaded into INMEX (http://www.inmex.ca/INMEX). [14] The data was annotated by converting different gene or Probe ID to Entrez IDs. For each probe set, intensity values were subjected to simple log 2 transformation followed by quantile normalization. The FDR was fixed at 0.01. After all the data-sets were uploaded, they were processed, annotated, and a data integrity check was performed prior to meta-analysis. The Cochran’s Q test was used to calculate the weighted sum of squared differences between individual study effects and pooled effect across studies. The QQ plot was observed and since the estimated Q values deviated significantly from the Chi squared distribution, random Effect Model with a significance of 0.05 was used for meta- analysis. The random effects model presumes that different studies present with substantial diversity, and evaluates the *between study variance* along with *within study sampling errors*. [15] The statistical analysis was done using the INMEX program.

### Functional Analysis

The Functional analysis of INMEX was used to generate a new hypothesis by exploiting the characteristics of the DEG in the meta-analysis. A heat map was created using the pattern extractor produced gene expression profiles across the different data-sets and conditions used for the study. A Gene Ontology (GO) enrichment analysis was performed using a web-based Software GENECODIS (http://genecodis.cnb.csic.es) to interpret the biological implications of the DEG in OTSCC. [16] GENECODIS integrated information from different sources like NCBI Entrez Gene, KEGG, Swiss-Prot and other databases for concurrent enrichment studies. Two statistical tests, namely, the hypergeometric distribution and the χ2 test of independence were applied to identify categories, and their combinations, that were significantly enriched in the list of genes. In addition, we also performed the pathway enrichment analysis based on the Kyoto Encyclopedia of Genes and Genomes (KEGG) database.

### Network construction

The protein-protein interactions (PPIs) were derived using GeneMANIA (www.genemania.org). [17] GeneMANIA created a consensus network of the gene of interest based of the top 30 up regulated and down regulated genes based on the genomic networks available in public available sources like Reactome, BioCyc, BioGRID, Pathways common. Here we studied the co-expression, physical interaction, genetic interaction, shared protein domains, co-localization, pathways involved and predicted functional relationships between the differentially expressed genes relevant to OTSCC. We also used STRING (www.string.db.org) for network construction. [18] STRING database has known and predicted protein interaction. The interactions include direct (physical) and indirect (functional) associations that are derived from four sources namely genomic context, high throughput experimentation, previous knowledge, conserved co-expression. STRING quantitatively integrated interaction data from these sources to derive the integration maps.

### Ethical Statement

All research involving human participants had been approved by the authors’ Institutional Review Board (IRB) and all clinical investigations had been conducted according to the principles expressed in the Declaration of Helsinki. A written informed consent was obtained from all the participants and the content of the informed consent was approved by the respective Institutional Research Boards namely, **Cancer Institute WIA; Protocol 1 HNCOG** (Cancer Institute, **W**omens **I**ndia **A**ssociation; Protocol 1 **H**ead and **N**eck **C**o- operative **O**ncology **G**roup); **SBDCECM105/13/58** (**S**ree **B**alaji **D**ental **C**ollege **E**thical **C**ommittee **M**eeting 105/13/158) and **IHEC/SD/MDS/120MP2** (**I**nstitute **H**uman **E**thics **C**ommittee/ **S**aveetha **D**ental/**M**aster of **D**ental **S**urgery/120**M**axillofacial **P**athology 2). The finger prints were obtained for patients who were illiterate after explaining the protocol and a written consent was additionally taken from patient’s relative presenting as witness.

### Patient Data

Retrospective samples used for the study were from paraffin embedded sections of early staged tongue cancers (clinical stages, T1 and T2 with N0) (n = 167) from patients presenting between the years 1995 and 2007 to the Head and Neck Oncology clinic, Cancer Institute (WIA). The prospective primary tongue cancer samples (n = 21), corresponding adjacent apparent normal tissue (n = 4) were obtained from the patients presenting to the Head and Neck Oncology clinic, Cancer Institute (WIA) and taken for wide excision glossectomy. Histologically normal tongue tissues (n = 4) and formalin fixed paraffin embedded tongue normals (n = 10) were obtained from patients presenting to the dental outpatient clinic of Sree Balaji Dental College and Hospital and Saveetha Dental College and Hospital. These patients were non tobacco users and presented with erythema on tongue suspected clinically as erythroplakia due to irritation. After biopsy, these samples were found histologically normal. All the prospective samples were collected and stored in RNA later. Variables recorded and evaluated for the study included age, sex, site, pattern of the lesion, depth of invasion, clinical stage, histological grade (both Broders grading and Bryne’s grading), occult node positivity and tobacco habits.

### Treatment Details

Comprehensive history and physical examination of the oral cavity and additionally upper aero-digestive tract with neck imaging was done using ultrasound for the OTSCC patients. The patients had undergone standard treatment at Cancer Institute (WIA) consisting of either wide excision glossectomy or brachytherapy, with or without selective neck dissection (Levels I to IV). Patients unwilling/unfit for surgery were treated using External Beam Radiotherapy as per the decisions of multidisciplinary board of the Institution. Pattern of Failure and good outcome was recorded for each patient.

### Histopathological analysis

Each patient had undergone a routine evaluation which included a biopsy for histological confirmation of cancer. All the clinical samples used for the validation studies (n = 206) were individually examined by two Oral pathologists (VS and PR) to assess the histopathology along with presence of percentage of tumor cells. Only tissues showing percentage of tumor cells >70% were included in validation studies. The invasive tumor front grading was done on OTSCC H and E stained slides according to criteria of degree of keratinisation, nuclear pleomorphism, pattern of invasion and lymphoplasmacytic infiltrate giving the scores from 1–4 as Bryne’s grade and invasive pattern grading score (IPGS) 1–8 as described previously. [19, 20] When true ITF was absent in the section, the deepest portion of the visible tumor was graded. The depth of invasion was measured from the highest portion of the basement membrane to the deepest portion of the tumor as described before [21] directly in micrometers using ProgRes CapturePro 2.8.8 software (JENOPTIK optical systems) at 4x objective magnification.

### Real time PCR

Real time PCR was performed on prospective tissue specimens (n = 29) to measure the mRNA expression of MMP9 and E-cadherin. The primer sequences used for the study are shown in Table 1. The quantitative real-time RT-PCR was performed using FastStart Universal SYBR Green Master (Rox) (Roche) according to the manufacturer’s instructions on a 7500 Real Time PCR System (Applied Biosystems). Universal thermal cycling conditions used were as follows: 10 min at 95°C, 40 cycles of denaturation at 95°C for 15 sec, and annealing and extension at 60°C for 1 min. Data was collected at every temperature phase during each cycle. The comparative threshold cycle (Ct) method was used to calculate fold change. β-Actin gene was used as a reference control to normalize the expression values. Triplicate reactions were performed for each gene expression studies and mean expression value was computed for subsequent analysis. The relative expression level of the genes was calculated using (2- ddct) method.

**Table 1:** Primers used for Real-time quantitative PCR.

Primer Name	Primer Sequence (5’– 3’)	Accession	Product Size (bp)
β-Actin For	GAGCACAGAGCCTCGCCTTT	NM_001101.3	108
β-Actin Rev	ACATGCCGGAGCCGTTGTC		
E-cadherin For	TTCCTCCCAATACATCTCCC	NM_004360	142
E-cadherin Rev	TTGATTTTGTAGTCACCCACC		
MMP9 For	CTTTGACAGCGACAAGAAGTGG	NM_004994.2	111
MMP9 Rev	GGCACTGAGGAATGATCTAAGC		

### Immunohistochemistry

The IHC detection of MMP9 and E-cadherin expression was performed on 5 μm sections of FFPE tissues (n = 167) and histologically normal sections (n = 10). The sections were deparaffinised in xylene and rehydrated in absolute ethanol. Antigen retrieval was done with 0.05M Tris Buffer (pH 9) in pressure cooker for 20 minutes. Endogenous peroxidase activity was blocked by incubation in 0.03% hydrogen peroxide in distilled water for 10 minutes and then washed with phosphate buffered saline (PBS). Sections were pre-incubated with power block (BioGenex Laboratories, San Ramon, CA) for 10 minutes and then incubated with primary antibody against MMP9 (BioGenex Cat#AN504, clone EP1255Y, rabbit monoclonal antibody) and E-cadherin (BioGenex Cat#AM390, clone 36, mouse monoclonal antibody) at room temperature for 90 minutes. MMP9 and E-cadherin expression was observed using the SuperSensitive™ Polymer-HRP IHC Detection System (BioGenex Laboratories, San Ramon, CA). Sections were counterstained with hematoxylin, dehydrated, and mounted in DPX. Positive controls and negative controls were included appropriately for MMP9 and E-cadherin where primary antibody was replaced with 2% BSA in negative control. Immunostaining of the sections was reviewed with the corresponding haematoxylin and eosin stained sections.

#### Scoring of MMP9 and E-cadherin

The immunohistochemically stained tissue sections were reviewed and scored independently by two Oral Pathologists VS and PR, blinded to the clinical parameters. For MMP9 scores, the staining intensity was scored as shown previously. [22, 23] The extent of MMP9 staining was defined as the percentage of positive staining areas of tumor cells or normal tongue epithelial cells in relation to the whole tissue area, scored on a scale of 0 to 4 as follows, 0, <10%; 1, 10–25%; 2, 26–50%; 3, 50–75%; 4, >76%. The sum of the staining intensity and the staining extent scores was used to calculate the final staining score. For statistical analysis, final staining scores of 0–5 and 6–7 were considered to be low and high MMP9 expression values respectively. E-cadherin was scored as described before. [24] The expressions were scored at the representative areas of mid tumor region as well as in invasive tumor fronts separately.

### Statistical Analysis

All statistical analyses were done in SPSS version 16.0. Distribution of categorical variables was compared by Pearson’s Chi-squared test or Fischer’s exact test according to the counts of expected frequencies. Overall Survival (OS) in months was calculated from the start of the treatment date to the last follow up date or alternately, date of death due to disease. Time in months to disease recurrence from start of treatment date was used to calculate disease free survival (DFS). Estimated survival curves were calculated by Kaplan Meier method and the results were compared using log rank test. To analyze the prognostic factors for the risk of recurrence and death, the patient groups and clinico-pathological characteristics were evaluated for association with time to recurrence and death using the Cox proportional hazards regression model. A hazard ratio (HR) with 95% confidence intervals (CI) from Cox model was obtained by univariate analysis. For multivariate analysis, the factors for which P- value was below 0.1 in univariate analysis were selected and model was developed based on enter method to derive significant prognostic variables. Statistical significance was given to the P values <0.05.

## Results

### Studies included in OTSCC Meta-analysis

**Fig 1:**
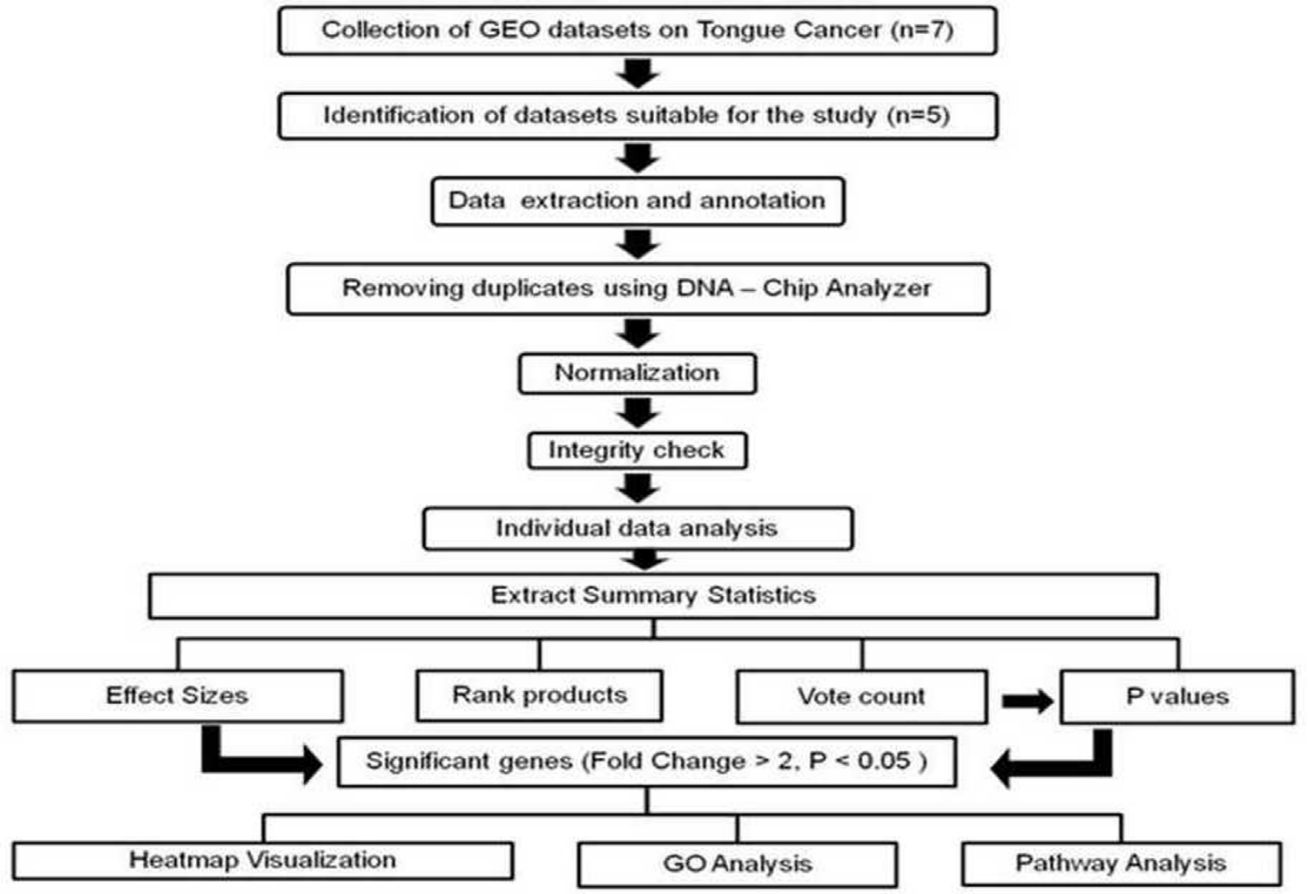
Flow chart representing the meta-analysis study design.

In the current study, we collected a total of 5 expression profiling study data-sets according to the inclusion and exclusion criteria specified, comprising of 111 tongue tumors and 79 normals serving as controls. Selected details of the individual data-sets are summarized in Table 2. The current study design is shown as a flow chart. (Fig 1)

**Table 2:** GEO Datasets used for the meta-analysis.

GEO ID	Samples (N/T)	Reference	Platform	Location
GSE9844	38 (12/26)	Ye et al., 2008 [25]	Affymetrix Human Genome U133 Plus 2.0 Array [HG-U133_Plus_2]	U.S.A.
GSE34106	43 (15/28)	Rentoft et al., 2012 [26]	Illumina HumanHT-12 WG-DASL V4.0 R2 expression beadchip	Sweden
GSE31056	46 (23/23)	Reis et al., 2011 [27]	Affymetrix GeneChip Human Genome HG-U133 Plus 2 Array [HG-U133_Plus_2]	U.S.A
GSE19089	6 (3/3)	Tuch et al., 2010 [28]	Illumina HumanHT-12 V3.0 expression beadchip	U.S.A
GSE13601	57 (26/31)	Estilo et al., 2009 [29]	Affymetrix Human Genome U95 Version 2 Array [HG_U95Av2]	U.S.A

### Class Comparison and common genes across databases by BRB array tools

The common genes across the 5 data-sets were derived by combined P-value method. Class comparison by BRB array tools gave a list of 434 DEG, of which 194 were up-regulated and 241 were down-regulated. (S1 Table) The advantage of this method is that the actual fold-change of gene expression and the consistencies of deregulation across the 5 datasets were also taken into consideration, which helped us to narrow down on genes that are both biologically and statistically significant.

### Individual data analysis followed by Meta-analysis of Gene Expression in OTSCC

**Fig 2:**
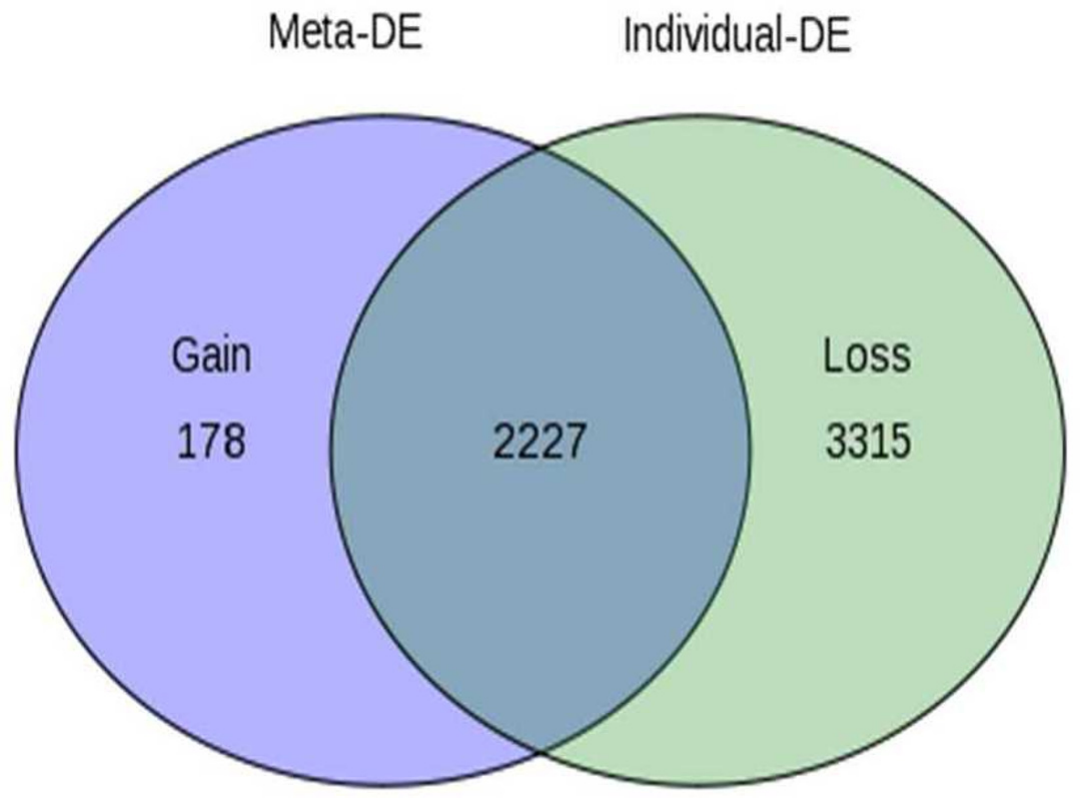
Graphical representation of the number of DEG from the meta-analysis.

The individual datasets were subsequently loaded into INMEX, and subjected to simple log 2 transformation and quantile normalization with a fixed FDR of 0.01. A random effects model of Effect Sizes (ES) measures was applied showing the integration of the gene expression patterns across the studies. The DEG with a significance of <0.05 were selected. The total number of DEG from the meta-analysis is illustrated as a Venn diagram in Fig 2. The ‘gained’ genes (n = 178) are the DEG identified in the current meta-analysis only and the ‘lost’ genes (n = 3315) are genes identified as DEG in any of the individual data analysis but not in the current meta-analysis These are the genes that present conflicting changes in the expression profiles or show large variations in different studies.

**Fig 3:**
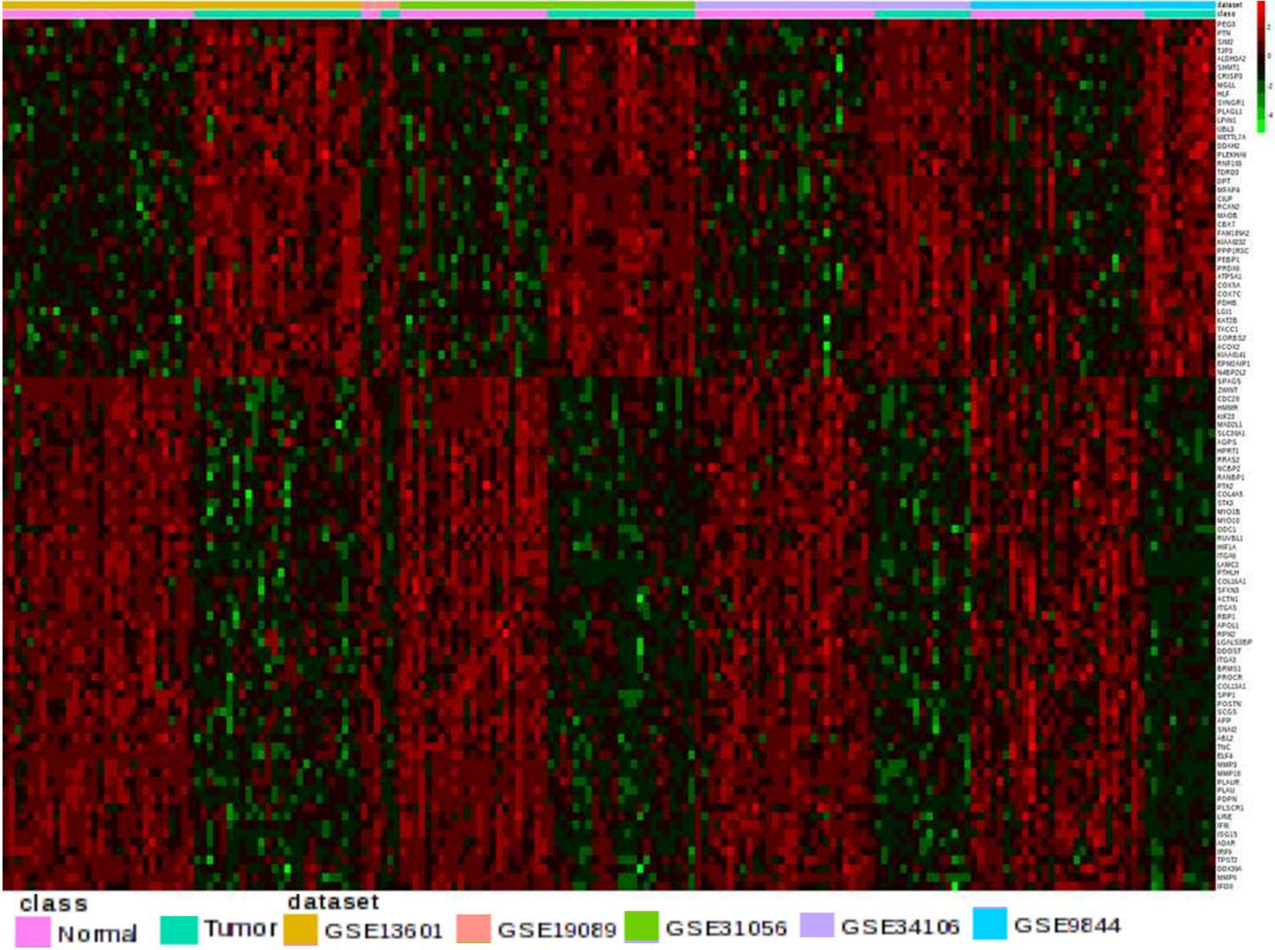
Heat map showing DEG. The up regulated (red) and down regulated genes (green) shown after meta-analysis. The top bar shows the GEO dataset ID and the second bar shows the classification of tumor vs. normal. The gene list is shown in the right.

Heat map showing hierarchical clustering depicts the total number of DEG (n = 2405) in OTSCC datasets. (Fig 3) There was a significant overlap in the DEG obtained by both the above methods. The top 30 up regulated and top 30 down regulated are shown in Tables 3 and 4. The S2 Table shows the full list of DEG categorized by combined ES and significant P value.

**Table 3:** Top up-regulated genes in OTSCC compared to normal.

Entrez Id	Gene Name	Combined ES score	P value
3918	LAMC2	2.7336	0.00
4321	MMP12	2.7026	1.26E^-06^
4430	MYO1B	2.6354	0.00
5744	PTHLH	2.5782	2.72E^-13^
9636	ISG15	2.5021	0.00
4312	MMP1	2.4509	2.88E^-10^
5754	PTK7	2.3551	7.44E^-06^
5328	PLAU	2.3292	0.00
10630	PDPN	2.2647	5.07E^-12^
4322	MMP13	2.1188	3.03E^-08^
4651	MYO10	2.0692	0.00
6696	SPP1	2.0656	0.00
2305	FOXM1	2.0478	7.46E^-07^
9447	AIM2	2.0361	4.80E^-06^
2537	IFI6	2.0184	0.00
4319	MMP10	1.9907	0.00
3371	TNC	1.9856	0.00
6591	SNAI2	1.9634	0.00
3429	IFI27	1.9537	2.73E^-09^
1288	COL4A6	1.9313	4.37E^-08^
7045	TGFBI	1.924	2.10E^-07^
4314	MMP3	1.9053	0.00
3655	ITGA6	1.8967	0.00
5329	PLAUR	1.895	0.00
3624	INHBA	1.8929	0.006304
4318	MMP9	1.8794	0.00
1687	DFNA5	1.877	1.21E^-07^
9805	SCRN1	1.8724	0.0012623
1001	CDH3	1.8628	7.67E^-05^
3430	IFI35	1.8592	3.11 E^-07^

**Table 4:** Top 30 down-regulated genes in OTSCC compared to normal.

Entrez ID	Gene Name	Combined ES score	P Value
3131	HLF	-2.1969	0.00
11170	FAM107A	-2.1871	0.00017041
23171	GPD1L	-2.1232	9.38E^-10^
23328	SASH1	-2.1219	2.90E^-09^
9413	FAM189A2	-2.0904	0.00
4129	MAOB	-2.0519	0.00
2012	EMP1	-2.0377	0.004744
8991	SELENBP1	-2.0319	0.00024964
9145	SYNGR1	-1.9467	0.00
7123	CLEC3B	-1.9228	0.0020399
58528	RRAGD	-1.9067	7.54E^-09^
11343	MGLL	-1.9019	8.87E^-12^
2053	EPHX2	-1.8859	1.49E^-05^
84525	HOPX	-1.8813	0.013522
4258	MGST2	-1.83	7.15E^-09^
8909	ENDOU	-1.805	0.020125
1675	CFD	-1.8041	8.08E^-05^
23242	COBL	-1.801	0.0032195
5549	PRELP	-1.8009	1.87E^-09^
2949	GSTM5	-1.7977	3.63E^-06^
5507	PPP1R3C	-1.7834	9.67E^-12^
66002	CYP4F12	-1.7735	2.99E^-09^
22849	CPEB3	-1.741	4.18E^-08^
5037	PEBP1	-1.7342	0.00
3248	HPGD	-1.7071	2.33E^-07^
5412	UBL3	-1.707	0.00
25840	METTL7A	-1.6913	0.00
1577	CYP3A5	-1.6826	1.32E^-09^
443	ASPA	-1.6796	0.0022601
224	ALDH3A2	-1.6764	0.00

### Functional Annotation of DEG in OTSCC

**Fig 4:**
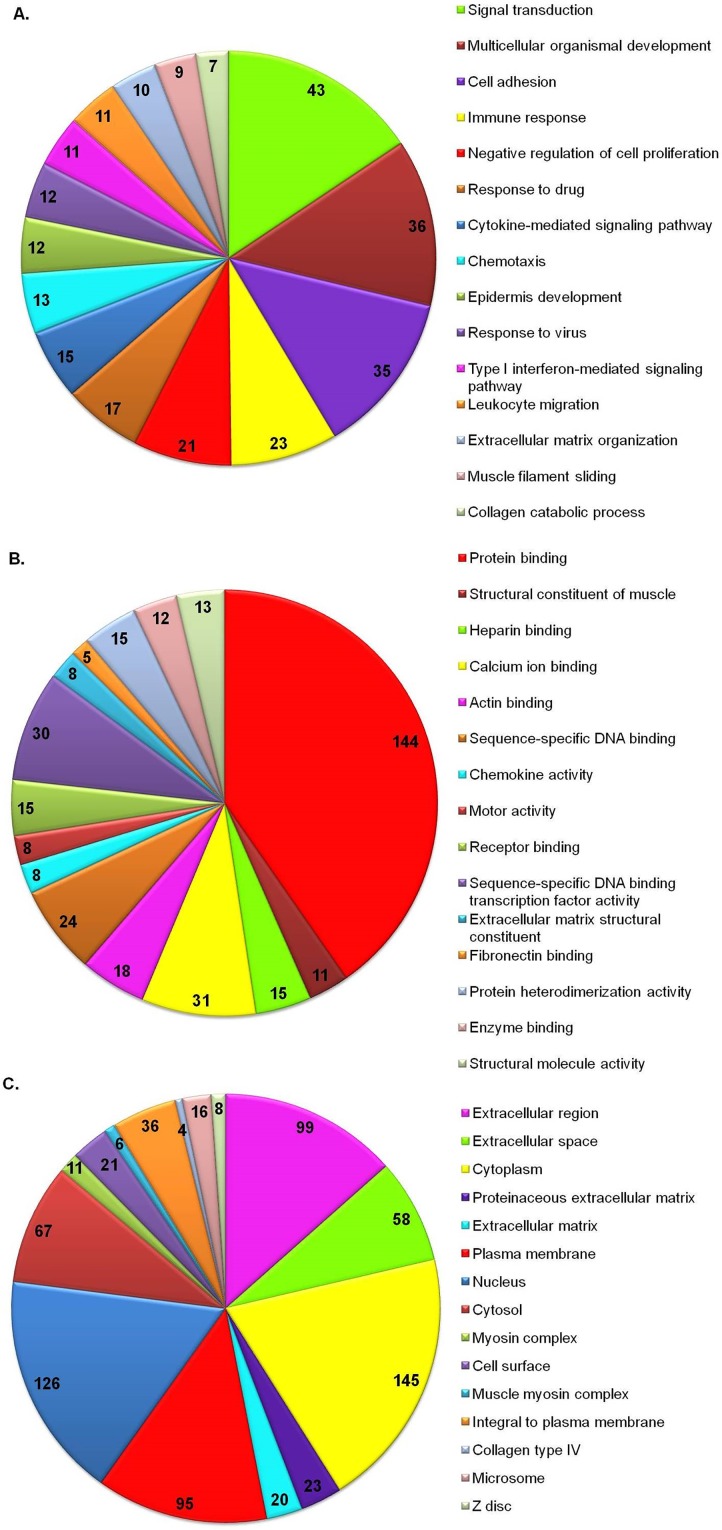
**Top 15 enriched GO terms of DEG** (A) Biological processes for DEG (P value = 3.57 E^-05^), (B) Molecular function for DEG (P value = 2.18E-^04^), (C) Cellular components for DEG (P value = 1.43E^-05^). The numbers indicate the number of genes per annotation. The numbers in pie-chart indicate the total number of annotated DEG for the respective terms.

To gain insights into the biological roles of the DEG from OTSCC, we performed a GO categories enrichment analysis. Gene ontology provides a common descriptive framework and functional annotation and classification to analyze the gene sets data. GO categories were organized into three groups: biological process, cellular component, and molecular function (Fig 4A, 4B and 4C). We found GO terms for Biological process enriched for Collagen Catabolic Process (GO:0030574; adj P value = 3.23 E-^07^), Extracellular matrix Disassembly (GO:2022617; adj P value = 1.74 E-^06^), response to stress (GO:0006950; adj P value = 5.29 E-^06^), Cellular component Movement (GO:0006928; adj P value = 9.12 E^-06^), and Biological Adhesion (GO:0022610; adj P value = 9.12 E^-06^) significantly enriched. We found GO terms for Molecular function enriched for metallopeptidase activity (GO: 0004222; adj P value = 2.55). S3 Table shows the Significantly Enriched GO processes of the top DEG.

#### OTSCC tumorigenesis involves breakdown of the extracellular matrix (ECM) mediated by up regulation of matrix metalloproteases (MMPs)

Several matrix metalloproteases were found to be up regulated in OTSCC namely MMP12, MMP1, MMP13, MMP10, MMP3 and MMP9 and they were among the top 30 up regulated genes involved in the aggressive course of OTSCC. (Table 3) It is well known that MMPs secreted by both the tumor and stromal cells are the chief players in ECM degradation promoting invasion and metastasis. The other important genes with related functions were LAMC2 (Combined ES = 2.7336), Myo1B (Combined ES = 2.6354) and Podoplanin (Combined ES = 2.2647).

#### Epithelial to mesenchymal transition (EMT) genes have important roles in OTSCC tumorigenesis

Cadherins CDH19, CDH16, CDH11 were found to be down regulated in OTSCC tumor as a read out of EMT process. Several genes implicated in EMT were found to be up regulated in OTSCC. Most of the integrins (ITGA5, ITGA2, ITGA5, ITGA2, ITGB6, ITGB4, ITGA3, and ITGA6) showed a combined ES value greater than 1.2 and notably GSK3beta also showed an up regulation. Tenacin (TNC), a known anti-adhesive ECM molecule and its known receptor integrin alpha beta 6 (ITGB6), which is important for assigning a mesenchymal property to the epithelial cell, was found to be up regulated. SNAI2/SLUG, an important gene in EMT process, was up regulated in OTSCC (Combined ES = 1.934). The encoded protein is well known to be involved in epithelial-mesenchymal transitions and also has anti apoptotic activity. SNAI2/SLUG acts as a transcriptional repressor binding to E-box motifs and is known to represses E-cadherin transcription. We found down regulation of genes like CAV2, KRT19, MITF, and NUDT13 which are also important to drive the EMT pathways.

#### Protein-Protein Interaction Network shows the members of the uPA-uPAR System having important implications in OTSCC

**Fig 5:**
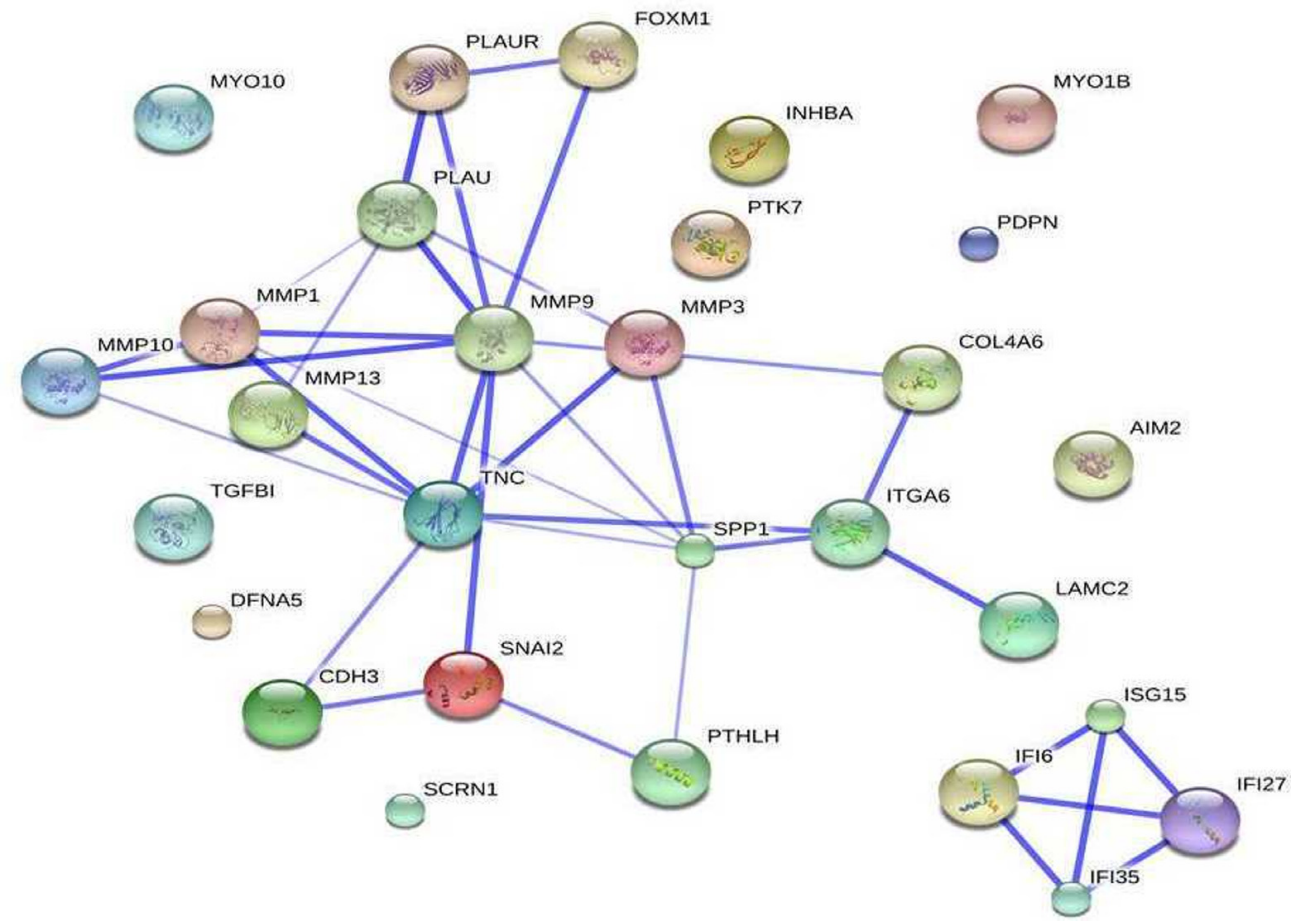
Protein-protein interaction network showing uPA and uPAR signaling pathway chiefly deregulated in OTSCC.

Construction of protein-protein interaction networks using the gene list obtained showed the uPA and uPAR system chiefly up regulated in OTSCC. (Fig 5) The PPI network also showed Osteopontin (SPP1), up regulated in OTSCC tumors directly interacting with uPAR. The urokinase plasminogen activator (uPA) system, consisting of genes uPA (PLAU) (Combined ES = 2.3292) and receptor uPAR (PLAUR) (Combined ES = 1.895) was found to be up regulated in OTSCC tumors compared to normals.

#### Collagen Catabolism pathway is activated in OTSCC

Several genes involved in collagen catabolism pathway were found to be differentially expressed in OTSCC, of which COL4A6, COL5A2 showed significant up regulation and COL14A1 showed down regulation.

#### The down regulated genes in OTSCC are implicated in detoxification of carcinogenic compounds and environmental toxins

Among the down regulated genes, Hepatic Leukemia Factor (HLF; Combined ES = -2.1969) encodes a member of the proline and acidic-rich (PAR) protein family, a subset of the bZIP transcription factors. The encoded protein forms homodimers or heterodimers with other PAR family members and binds sequence-specific promoter elements to activate transcription. OTSCC also showed down regulation of FAM107A, (Combined ES = -2.1871) a tumor suppressor gene and MGST2 (Combined ES = -1.83), a gene encoding a protein catalyzing the conjugation of leukotriene A4 and reduced glutathione to produce leukotriene C4, the important mediators of inflammation. The other important genes were GSTM5 (Combined ES = -1.7977), the mu class of GST enzyme, EPHX2 (Combined ES = -1.8859), enzyme with well known functions of detoxification of carcinogens and degradation of aromatic compounds to be excreted out of body were found to be down regulated in OTSCC tumors, probably increasing the susceptibility to toxicity due to exposure.

### Validation Studies

#### Quantitation of MMP9 and E-cadherin mRNA expression in OTSCC tumors

**Fig 6:**
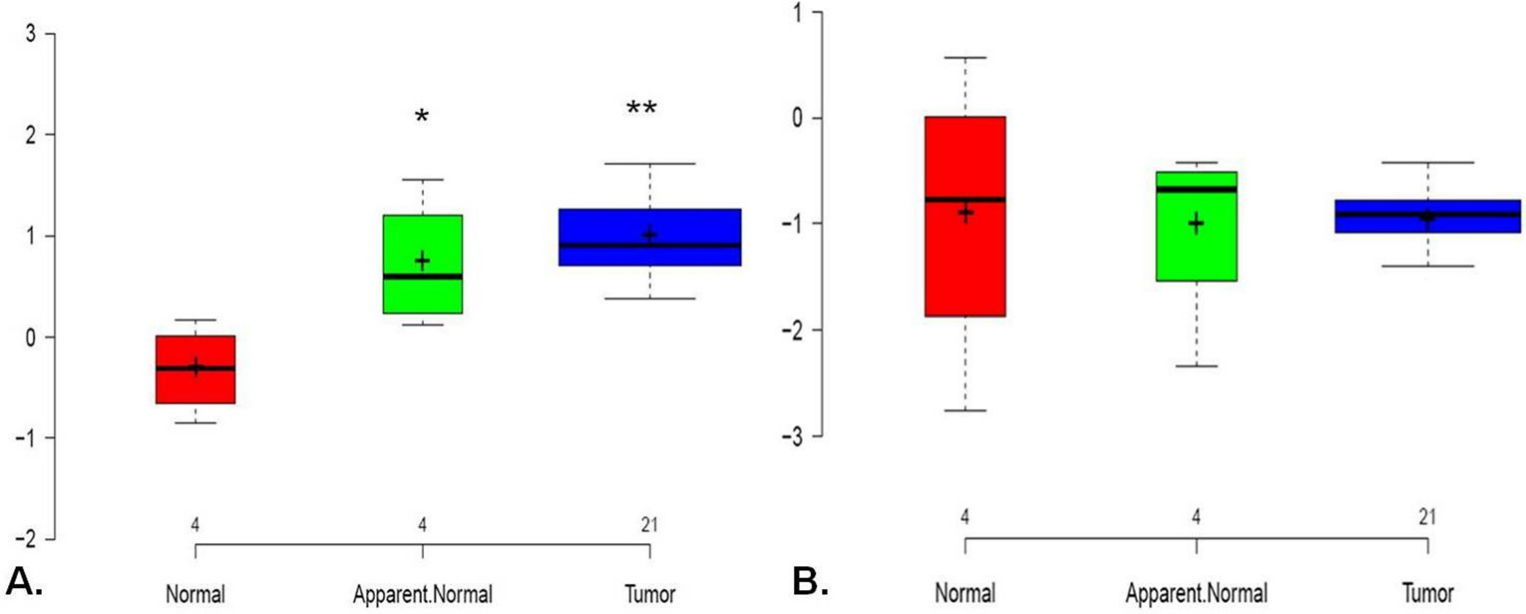
**Box-plot comparing the relative gene expression of (A) MMP9 and (B) E- cadherin in OTSCC normal, apparent normal and tumors**. Centre line shows the median and + indicates sample mean.

MMP9 mRNA was significantly up-regulated in OTSCC compared to apparent normal (**P value = 6 E-^05)^ and as compared to histologically normal tongue tissue. (* P value = 0.02) E-cadherin mRNA expression was found to be down regulated in tumors, however, it did not show a statistically significant deregulation comparing the pairs of tumors and normals (Fig 6)

#### MMP9 protein over-expression denotes poor prognosis and is significantly associated with disease recurrence and poorer DFS in OTSCC patients

**Fig 7:**
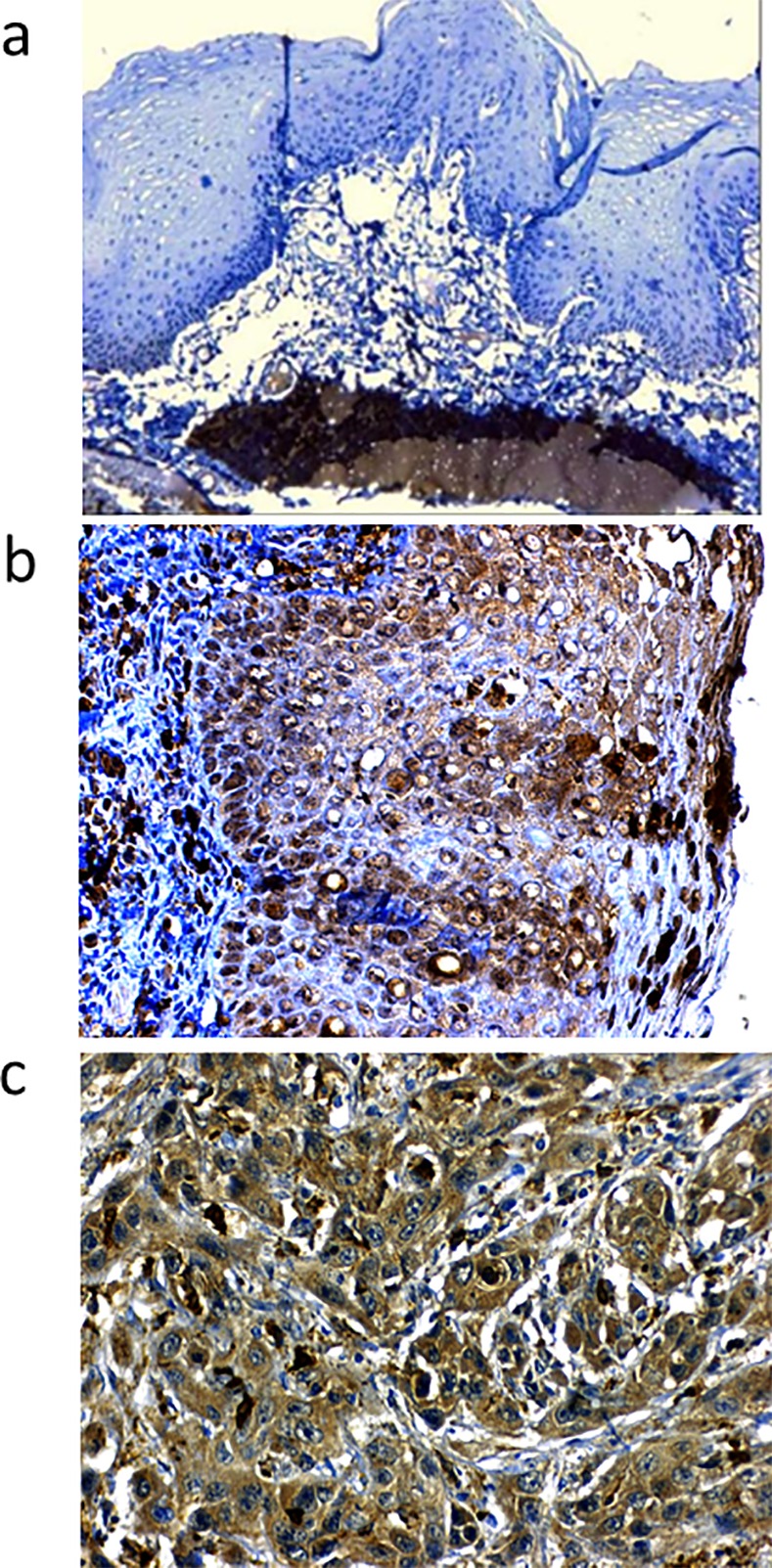
**MMP9 expression by IHC** (A) Normal tongue epithelium with no MMP9 expression (10X) (B) Intense MMP9 staining in areas showing severe dysplasia (40X) (C) Moderately differentiated SCC showing intense and diffuse MMP9 stain. (40X).

**Fig 8:**
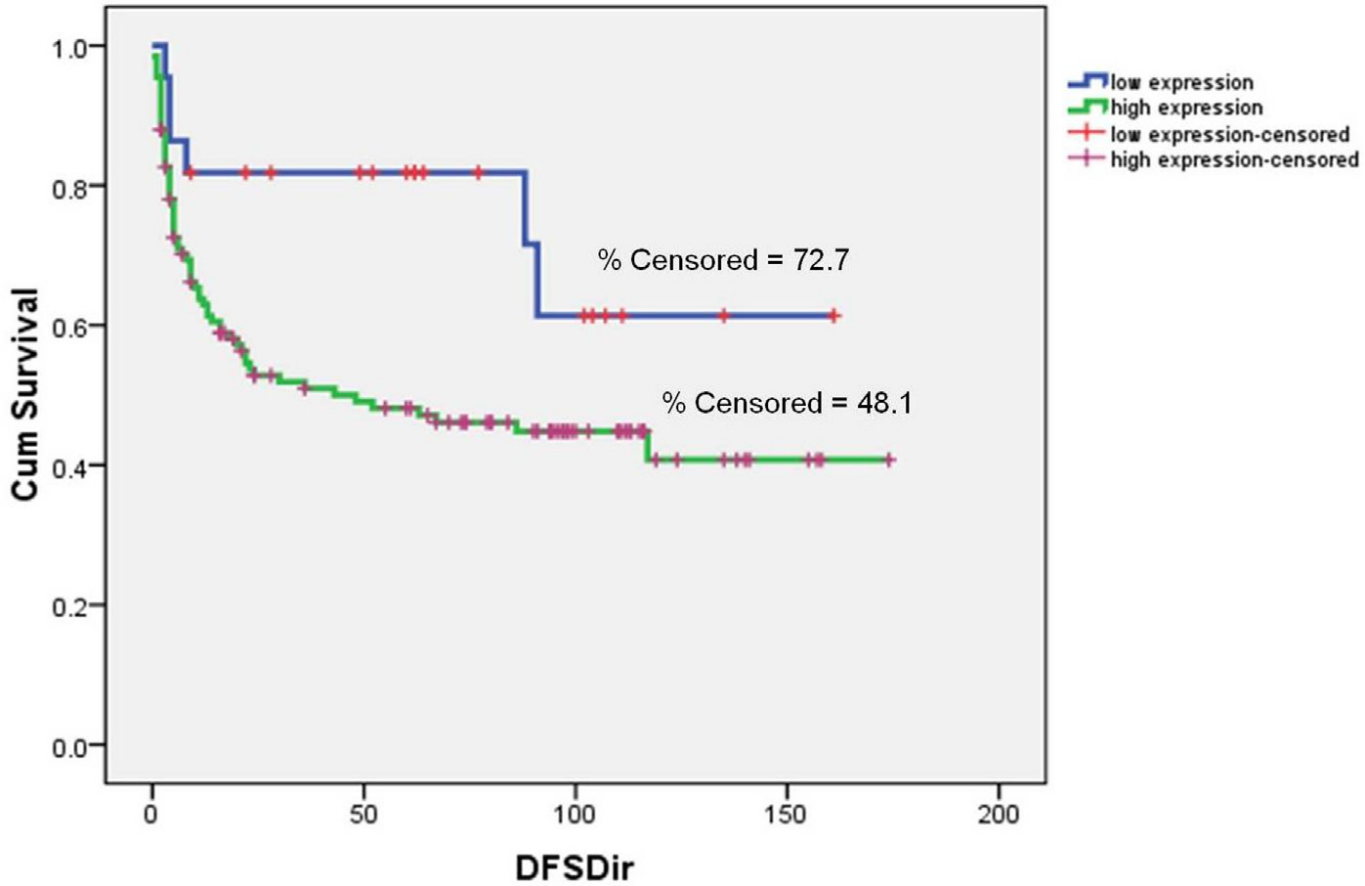
Kaplan-Meier survival curve for DFS based on level of MMP9 expression. **(A)** Upper blue line represents low MMP9 expression while lower green line indicates high MMP9 expression. The short vertical lines denote the times at which patients were lost to censoring. The difference in DFS between patients with high and low expression of MMP9 was statistically significant (Log rank = 4.779; P value = 0.029).

Immunohistochemistry showed normal tongue mucosa epithelium negative for MMP9 expression. Positive MMP9 staining was noted in muscle cells which served as internal positive control. The characteristic distribution pattern of MMP9 in our series was found to be diffuse expression in both the tumor and stromal areas of OTSCC. Fig 7A, 7B and 7C show the pattern of expression of MMP9 in normal, severe dysplastic areas and OTSCC tumors. Of the 167 OTSCCs stained for MMP9, 136/167 (81.4%) showed a high expression of the protein. Higher Bryne’s grade was significantly associated with over-expression of MMP9 (χ2 = 12.695;P value = 0.026). Also, MMP9 over expression was very significantly correlated with failure of treatment (χ2 = 12.609; P value = 0.000). Among the patients who failed, 87/96 (90.6%) had a high MMP9 expression in the respective OTSCC compared to 49/71 (69%) of OTSCC among patients who showed no evidence of disease. Over expression of MMP9 was found to be significantly correlated to pattern of recurrence (χ2 = 14.361; P value = 0.006). (Table 5) Among the patients who failed (n = 96), high expression of MMP9 was observed in 90.3% (28/31) with local recurrence, 100% (19/19) with nodal recurrence, 86.4% (38/44) with loco-regional recurrence and 100% (2/2) with distant metastasis. Evaluating the prognostic variables by univariate and multivariate Cox proportional hazard model, patients with MMP9 over expression had an increased hazard of poorer DFS (HR = 2.288; P value = 0.037 and HR = 2.259; P value = 0.044) respectively. (Tables 6 and 7) Kaplan Meier survival curves showed patients with low expression of MMP9 having a statistically significant (Log rank = 4.779; P value = 0.029) better disease free survival (DFS) (67.7%) compared to patients with over expression (47%). (Fig 8)

**Table 5:** MMP 9 expression vs Pattern of Recurrence.

Pattern of Recurrence	MMP9 high	MMP9 low
No evidence of disease	49(69)	22 (31)
Local Recurrence	28(90.3)	3(9.7)
Nodal Recurrence	19 (100)	0
Locoregional Recurrence	38(86.4)	6(13.6)
Distant metastasis	2 (100)	0

P value = 0.006; NED—No evidence of disease

**Table 6:** Univariate Cox Regression Analysis for prognostic variables.

Clinicopathological Factor	DFS			OS		
	P value	HR	95% CI	P value	HR	95% CI
Age< = 55 years> 55 years	0.295	0.774	0.480–1.249	0.571	1.165	0.685–1.979
Gender	0.628	1.120	0.708–1.774	0.078	1.512	0.954–2.397
Tobacco Habits	0.054	1.541	0.993–2.389	0.312	1.262	0.804–1.980
T stageT1T2	0.054	1.587	0.993–2.536	**0.013**	**1.866**	1.143–3.048
Grade	0.136	1.415	0.896–2.233	0.110	1.458	0.919–2.314
Tumor Depth< = 4mm>4mm	0.762	0.913	0.508–1.642	0.646	0.864	0.463–1.613
Occult Node Positive	**0.007**	0.230	0.079–0.671	0.832	0.846	0.179–3.991
Treatment Modality	0.767	1.054	0.746–1.490	0.240	1.223	0.874–1.713
Upfront neck Management	0.145	1.103	0.967–1.258	0.291	0.928	0.807–1.066
MMP 9 Over Expression	**0.037**	**2.288**	1.051–4.983	0.612	1.188	0.610–2.316
E-cad loss at ITF	**0.045**	**1.566**	1.011–2.426	0.072	1.468	0.966–2.230

Note: Statistically significant P values are shown in bold

**Table 7:** Multivariate Cox Regression Analysis for prognostic variables.

Clinicopathological Factor	DFS			OS		
	P value	HR	95% CI	P value	HR	95% CI
MMP 9 Overexpression	**0.044**	**2.259**	1.022–4.997			
E-cadherin Loss at ITF	0.06	1.142	0.993–1.314	**0.003**	1.224	1.069–1.402
Occult Node Positive	**0.019**	**0.211**	0.58–0.772			

Note: Statistically significant P values are shown in bold

#### Loss of membrane positive E-cadherin expression at invasive tumor front indicates failure and poorer DFS

**Fig 9:**
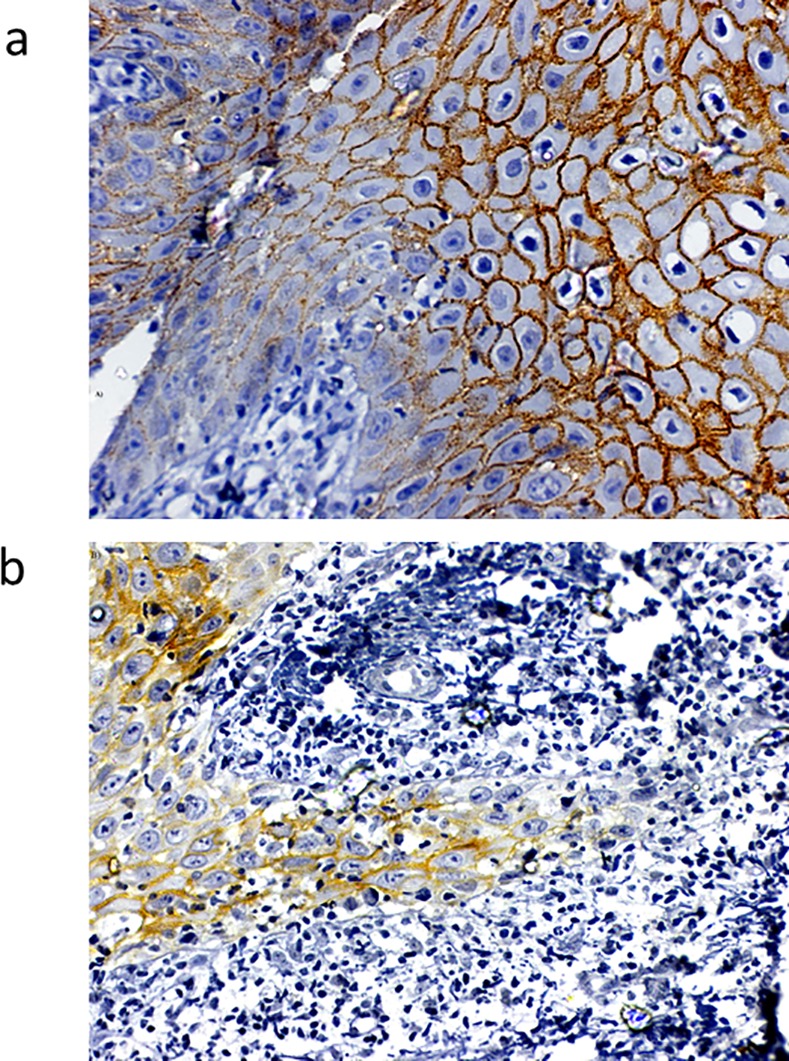
**Expression of E-cadherin by IHC** (A) Membrane staining of E-cadherin in OTSSC (40X) (B) Progressive reduction of E-cadherin positivity from the surface epithelium to invasive tumor front (40X).

**Fig 10:**
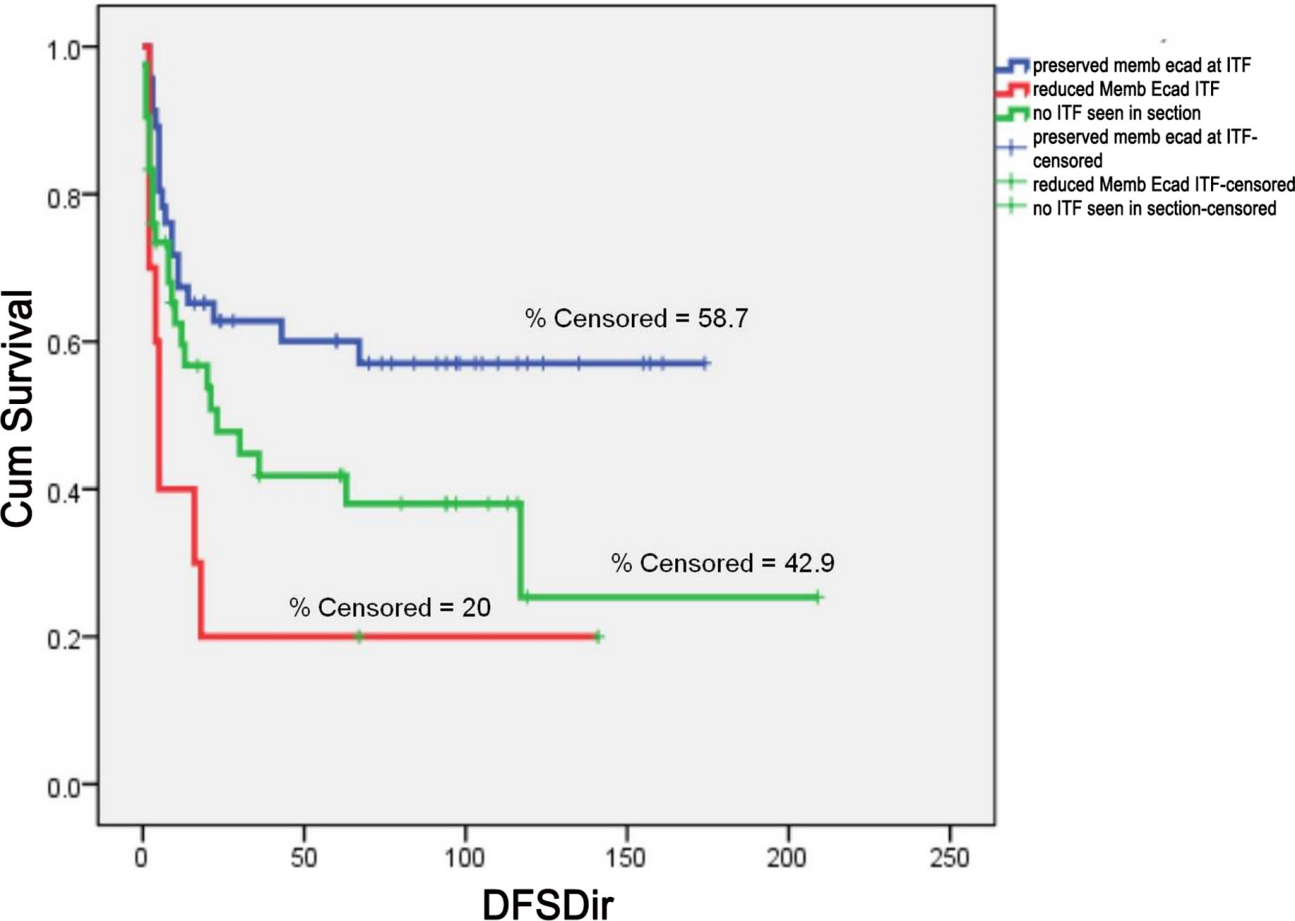
Kaplan-Meier survival curve for DFS based on E-cadherin expression at ITF. (A) First blue line indicates preserved membrane E-cadherin positivity at ITF, second green line indicates reduced membrane E-cadherin expression at ITF and third red line indicates absence of ITF in the sections. The short vertical lines denote the times at which patients were lost to censoring (Log Rank = 8.063; P value = 0.018).

E-cadherin staining results are reported for 156 patients with OTSCC. The staining was unfit for interpretation with folds and loss of tissue in 11 samples. Fig 9A shows the pattern of expression of E-cadherin in OTSCC. The membrane staining of E-cadherin was found to be preserved in 107/156 (68.5%) and staining was found to be absent for 26/156 (16.6%). Loss of membrane staining and displaced intense cytoplasmic staining for E-cadherin was found in 23/156 (14.7%). E-cadherin expression was significantly associated with differentiation of OTSCC tumors (χ2 = 10.024; P value = 0.007). Loss of membrane positivity of E-cadherin was positively correlated to higher IPGS scores (χ2 = 20.829; P value = 0.002). There was a significant higher nodal and loco-regional recurrence among patients whose tumors showed loss of E-cadherin membrane expression at the invasive tumor front of the OTSCC (χ2 = 14.115; P value = 0.028). Univariate and Multivariate analysis showed an increased hazard of poor DFS (HR = 1.566; P value = 0.045) and poor OS (HR = 1.224; P value = 0.003) respectively. (Tables 6 and 7) Kaplan Meier curves survival showed a significant poorer DFS (Log rank = 8.063; P value = 0.018) for patients showing loss of E-cadherin at ITF. (Fig 10)

#### Relevance of combined pattern of Expression for E-cadherin and MMP9 at ITF in OTSCC

**Fig 11:**
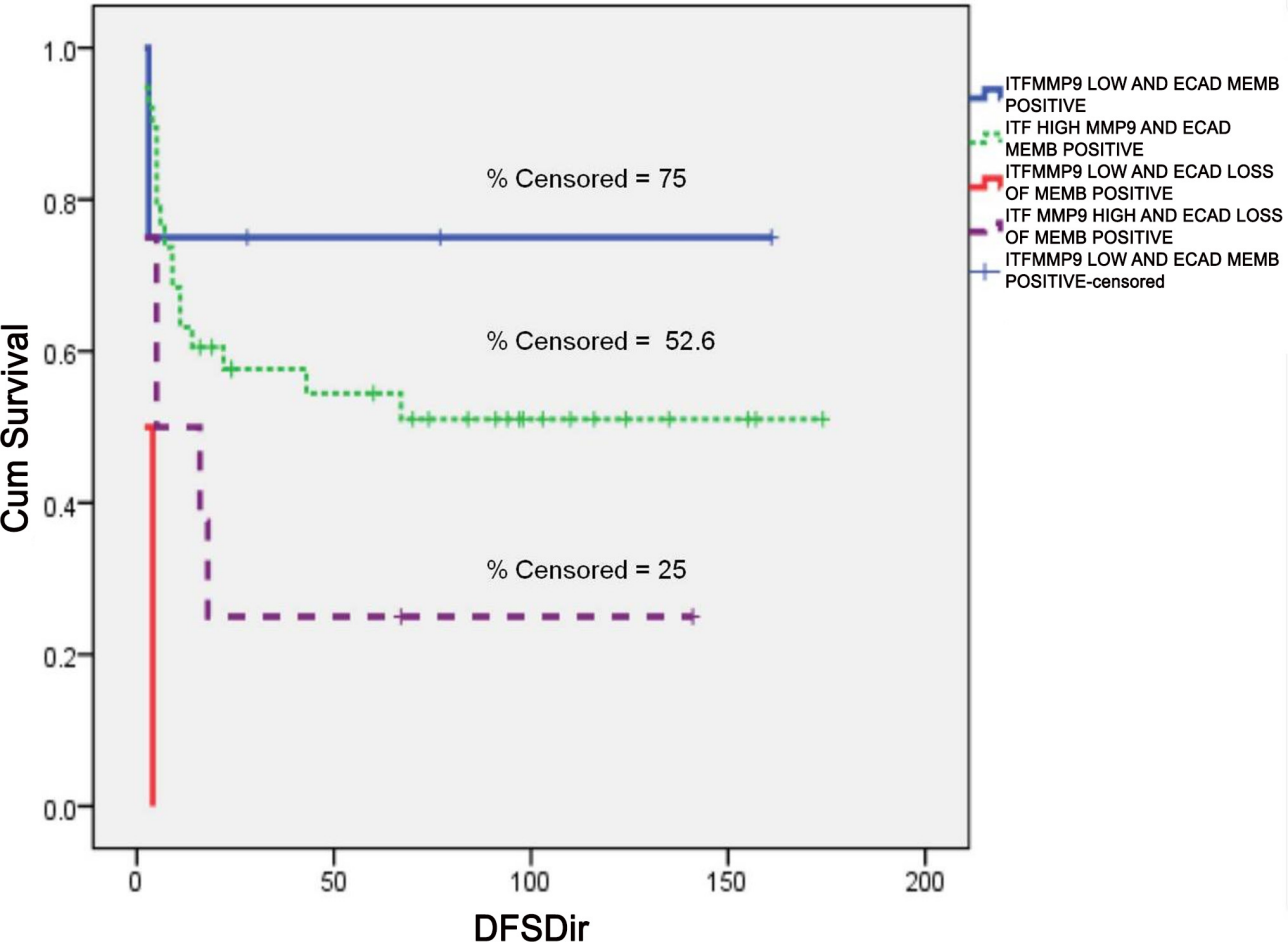
Kaplan-Meier curve for DFS based on E-cadherin and MMP9 expression at ITF. (A) First blue line indicates low MMP9 expression with expression of E-cadherin at membrane in ITF, second green line represents high MMP9 expression with expression of E-cadherin at membrane in ITF, third red line is indicative of low MMP9 expression with loss of E-cadherin expression at membrane in ITF, fourth purple line indicates high MMP9 expression with loss of E-cadherin expression at membrane in ITF. The short vertical lines denote the times at which patients were lost to censoring. Log Rank = 14.804; P value = 0.002.

There was a significant correlation (χ2 = 10.952; P value = 0.027) between intense MMP9 over expression and loss of membrane positive E-cadherin expression at invasive tumor front. (Table 8) Patients showing intense MMP9 expression combined with reduced membrane and increased cytoplasmic positivity of E-cadherin at the invasive front had the poorest DFS (Log Rank = 14.804; P value = 0.002). (Fig 11)

**Table 8:** E-cadherin expression at ITF vs MMP9 expression.

MMP9 ITF expression	E-cad Negative	Preserved E-cad Membrane Positive	Loss of Membrane Positive E-cad
Mild	22 (75.9)	5 (17.2)	2(6.9)
Moderate	12(46.2)	12(46.2)	2 (7.7)
Intense	22(39.3)	27(48.2)	7(12.5)
P = 0.027			

#### Other Prognostic Variables in OTSCC

Occult cervical node metastasis detected by elective neck dissection and a subsequent neck recurrence was observed in 17% of patients of which 30% belonged to stage T2. Univariate analysis showed tobacco habits, clinical stage tending to influence DFS, but OS was found be significantly associated with T stage (P value = 0.013). However, occult node positivity was found to be a significant predictor of poor DFS (P value = 0.007 and 0.019) by both univariate and multivariate Cox regression analysis models respectively along with the biomarkers discussed.

## Discussion

The current study is the first attempt of an integrated meta-analysis of OTSCC gene expression profiles with the primary objective of deriving a molecular portrait of tongue cancer. We show that meta-analysis can increase the statistical power towards a more precise estimate of gene expression, increasing the reliability, resolving inconsistencies, and reducing the likelihood of random errors. Our analysis showed loss of 3315 genes, indicating that these genes would have been otherwise deemed significant and pursued in individual studies but is not perceived as relevant when analyzed across the 5 datasets. A “gain” of 178 genes depicts the ones very significant in meta-analysis along with common genes that are consistent across the datasets that are more reliable and reproducible for unraveling the OTSCC biology. OTSCC has a poorer outcome with a survival rate of ~50% which has remained unchanged for the past four decades. [30] We have shown previously that OTSCC in Indian population has been showing a changing epidemiological trend with nearly 50% of the cases being non- tobacco associated [3] and also unlike oropharyngeal cancers, p16 expression was not found to be a surrogate marker for HPV in OTSCC. [31, 32] The rationale of the current study was therefore to understand the biology of the OTSCC using gene expression profiles with a larger sample size deriving the molecular aspects to help derive newer management strategies in future for this aggressive cancer.

### Tumor Microenvironment with MMPs, various ECM and EMT mediators in OTSCC

The molecular portrait derived in the current study shows OTSCC to be disease of tumor microenvironment. The top deregulated genes, are those involved in extracellular matrix remodeling and epithelial to mesenchymal transition (EMT) processes which can contribute towards an increased propensity of invasion leading to poorer outcome of OTSCC. About 6 members of the MMP family (MMP12, MMP1, MMP13, MMP10, MMP3, and MMP9) were among the top up regulated genes which can play significant role in all aspects of tumor progression via the enhancement of tumor-induced angiogenesis and the destruction of the local tissue architecture, thus allowing tumor invasion and metastasis. [33] Invasion of the tissues and metastasis to neck nodes is very common in OTSCC and is adverse in prognosis. In our current series also, occult node positivity was very significantly associated with a poor DFS. The MMPs are well known to facilitate ECM degradation, collagen breakdown, basement membrane degradation important for tumor cells to gain access to lymphatics and blood vessels, resulting in dynamic changes in the structure of the ECM, implying the aggressive course of the disease. [34] Some of the other relevant genes that are interesting to be pursued in future studies are LAMC2, MYO1b and podoplanin. LAMC2 (a major component of laminin-5) has been reported to be highly expressed in several types of invasive tumors [35, 36] including carcinomas of the pancreas [37, 38], tongue [39, 40], colorectal [41, 42], lung [43], cervix [44], lung [45], extra hepatic cholangiocarcinoma. [46] An elevated level of *LAMC2* in human cancers has been shown to be associated with poor survival in esophageal cancers. [47] Recent studies have shown that Myo1b is functionally involved in lymph node metastasis of human HNSCC. [48] Podoplanin identified in the current study has been reported as a reliable marker to determine the presence of lymphatic vessels, differentiating lymphatic endothelium from endothelium of blood vessels. Increased podoplanin has been shown to depict increased lymphatic vessel density indicative of lymph node metastasis. [49, 50, 51, 52] Earlier studies suggest that podoplanin mediates the remodeling of the actin cytoskeleton via the down regulation of the activities of small Rho family GTPases. [53] Therefore LAMC2, Myo1B and podoplanin can be further explored as useful markers for OTSCC prognosis.

### UPA-uPAR

Our data on protein-protein interaction network showed that uPA-uPAR system is chiefly involved in OTSCC tumorigenesis and suggests that it is an important pathway warranting more studies using relevant inhibitors. UPA-uPAR system has implications on tumor survival, growth, migration, invasion and metastasis. It is also well known that the activation of plasmin by cleavage of plasminogen is catalyzed by uPA binding to uPAR and is known to unleash a cascade of proteolysis by plasmin including the substrates MMP2 and MMP9 which can degrade the extracellular matrix. [54, 55]

### Detoxification pathways down regulated

Interestingly, the current study showed the genes down regulated, to be involved in xenobiotic metabolic pathways that are important for metabolism of carcinogens which may or may not be tobacco related. Our data showed FAM107A down regulation warranting a more detailed study in OTSCC in future. Studies have shown that FAM107A is dramatically decreased non-small cell lung cancers (NSCLC) with a minority of samples showing promoter methylation. [56] FAM107A has been described in renal cell carcinoma as a putative tumor-suppressor gene according to its role in the regulation of apoptotic processes [57, 58] and has been found in the cluster of hypermethylated and transcriptionally repressed genes in hepatocellular carcinoma. [59] Our studies also showed GSTM5, the mu class of GST enzyme, involved in the detoxification of electrophilic compounds including carcinogens, therapeutic drugs, environmental toxins and products of oxidative stress, to be down regulated. EPHX2 was another prominent down regulated gene identified in the current study which codes for Epoxide hydrolases which are important biotransformation enzymes. They convert epoxides obtained from the degradation of aromatic compounds to trans- dihydrodiols which can be conjugated and excreted from the body. Down regulation of these enzymes in OTSCC may have significant implications in inefficient degradation of epoxides to dihydrodiols. MITF, another down regulated gene in our data set functions as a ‘rheostat model’ controlling phenotypic switches between proliferative, differentiated and tumorigenic/invasive phenotypes as shown in malignant melanoma. [60]

### Validation of MMP9 and E-cadherin

Conventional histopathology lacks the power to discriminate between lesions that can recur compared to those that will not, therefore having little efficacy in prognostication of early staged tongue cancers. Our study showed patients with MMP9 over expression having a poorer DFS, very highly indicative of lymphatic metastasis consistent to earlier reports. [61, 62, 63, 64, 65] Well known EMT marker E-cadherin, being a membrane adhesion protein, showed preserved activity in the membrane and indicated aberrant expression when observed in cytoplasm. Current study showed loss of E-cadherin at ITF indicated poorer prognosis as shown previously. [66,67,68] The current study suggests that pattern of MMP9 and E-cadherin protein expression both in the centre and superficial areas of the OTSCC tumor and ITF can predict the treatment outcome, as evaluated by immunohistochemisty. It is well known that transformation of the cohesive and polarized epithelial cells into mesenchymal like cells confer the high mobility mark the hall mark of EMT. [69, 70, 71, 72, 73] Since the deepest and most invasive areas of tumor are histologically located at the ITF, these areas are known to orchestrate the clinical behavior. [19,74] Thus combined MMP9 over expression and loss of E-cadherin in OTSCC imply the aggressive nature of the disease.

The limitation of the current study attempted as a meta-analysis exercise is that all the samples studied belonged to a particular ethnic group, mostly Americans. So the results may vary marginally in other ethnic groups like Asians, among whom OTSCC is more prevalent. Still, the present study has been able to provide a comprehensive view of the biology of OTSCC along with the relevant genes and pathways to be pursued.

## Conclusion

Current study indicates that ECM is severely compromised and EMT processes are activated in OTSCC. The key genes involved in degradation of basement membrane and lamina propia, and EMT activation can lead to infiltration and metastasis, explained by activated collagen catabolism pathways. Down regulation of genes involved in xenobiotics of the carcinogens should also be probed further for mechanisms. The probable “triggers” for these conditions have to be explored in detail. Validation of Meta data showed that prognostic implications for OTSCC can be derived by evaluating E-cadherin and MMP9 expression at the invasive tumor front by a routine technique like immunohistochemistry. The possibility of exploring the given markers in liquid biopsies can also help in depicting prognosis and detecting residual disease post treatment. Newer clinical application of agents that can inhibit the mediators of ECM degradation may be a key to achieving clinical control of invasion and metastasis of tongue squamous cell carcinoma.

## Supporting Information

S1 Table Combined P values across the 5 datasets by BRB array tools. (XLSX)

S2 Table INMEX meta-analysis based on Combined Effect size and corresponding P values. (XLS)

S3 Table Gene Ontology analysis using GENECODIS. (XLSX)

S1 File PRISMA flow diagram. (DOC)

S2 File PRISMA Checklist. (DOC)
