# Supplementary material for: Molecular Portrait of Oral Tongue Squamous Cell Carcinoma Shown by Integrative Meta-Analysis of Expression Profiles with Validations
Source: PLoS One. 2016 Jun 9;11(6):e0156582. doi: 10.1371/journal.pone.0156582 (PMC4900586; doi:10.1371/journal.pone.0156582)
Supplement: S1 File — (DOC) [file pone.0156582.s004.doc]

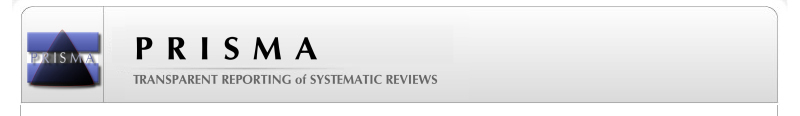
**S1 File : PRISMA 2009 Flow Diagram**

**Screening**

**Included**

**Eligibility**

**Identification**

Records identified through database searching
(n = 88 )

Additional records identified through other sources
(n =0 )

Records after duplicates removed
(n = 15 )

Records screened
(n = 15 )

Records excluded
(n = 10 )

Full-text articles assessed for eligibility
(n = 7 )

Full-text articles excluded, with reasons – Only tumor with no normals, paraffin samples
(n = 2 )

Studies included in qualitative synthesis
(n =5 )

Studies included in quantitative synthesis (meta-analysis)
(n = 5 )
